# Quality Assessment and Validation of High-Throughput Sequencing for Grapevine Virus Diagnostics

**DOI:** 10.3390/v13061130

**Published:** 2021-06-11

**Authors:** Nourolah Soltani, Kristian A. Stevens, Vicki Klaassen, Min-Sook Hwang, Deborah A. Golino, Maher Al Rwahnih

**Affiliations:** 1 Department of Plant Pathology, University of California-Davis, Davis, CA 95616, USA; nosoltani@ucdavis.edu (N.S.); dagolino@ucdavis.edu (D.A.G.); 2 Foundation Plant Services, University of California-Davis, Davis, CA 95616, USA; kastevens@ucdavis.edu (K.A.S.); vaklaassen@ucdavis.edu (V.K.); mshwang@ucdavis.edu (M.-S.H.); 3 Department of Computer Science, University of California-Davis, Davis, CA 95616, USA; 4 Department of Evolution and Ecology, University of California-Davis, Davis, CA 95616, USA

**Keywords:** high-throughput sequencing, grapevine virus, validation, performance assessment, sensitivity, specificity, reproducibility, repeatability

## Abstract

Development of High-Throughput Sequencing (HTS), also known as next generation sequencing, revolutionized diagnostic research of plant viruses. HTS outperforms bioassays and molecular diagnostic assays that are used to screen domestic and quarantine grapevine materials in data throughput, cost, scalability, and detection of novel and highly variant virus species. However, before HTS-based assays can be routinely used for plant virus diagnostics, performance specifications need to be developed and assessed. In this study, we selected 18 virus-infected grapevines as a test panel for measuring performance characteristics of an HTS-based diagnostic assay. Total nucleic acid (TNA) was extracted from petioles and dormant canes of individual samples and constructed libraries were run on Illumina NextSeq 500 instrument using a 75-bp single-end read platform. Sensitivity was 98% measured over 264 distinct virus and viroid infections with a false discovery rate (FDR) of approximately 1 in 5 positives. The results also showed that combining a spring petiole test with a fall cane test increased sensitivity to 100% for this TNA HTS assay. To evaluate extraction methodology, these results were compared to parallel dsRNA extractions. In addition, in a more detailed dilution study, the TNA HTS assay described here consistently performed well down to a dilution of 5%. In that range, sensitivity was 98% with a corresponding FDR of approximately 1 in 5. Repeatability and reproducibility were assessed at 99% and 93%, respectively. The protocol, criteria, and performance levels described here may help to standardize HTS for quality assurance and accreditation purposes in plant quarantine or certification programs.

## 1. Introduction

Grapevine, *Vitis vinifera*, hosts several disease agents, including over eighty-six virus species [1], which are readily transferred between vines through vectors and propagation materials. The economic loss due to virus infection negatively impacts grapevine industries, nurseries, and growers. For example, in California, estimates of the economic damage caused by grapevine leafroll-associated 3 (GLRaV-3) as the major virus involved in grapevine leafroll disease are more than $90 million annually [2,3]. Grapevine red blotch disease also causes more than $69,500 in damages per hectare over the predicted 25-year life span of a vineyard [4,5].

The cornerstone of sustainable production of specialty crops including grapevines is certification programs, such as the California Grapevine Registration & Certification (R&C) Program. This program targets the elimination of specific grapevine viruses that are spread through grafting/propagation. It is administered by the California Department of Food and Agriculture (CDFA) and Foundation Plant Services (FPS), the largest nationally recognized program that provides plant importation, quarantine services, virus testing, and virus elimination. Vines that are destined to enter the CDFA R&C program are tested, or are propagated from sources that have been tested, using methods prescribed in the CDFA regulations for foundation stock. These methods include herbaceous and woody indexing (bioassay) in addition to molecular testing methods such as the polymerase chain reaction (PCR). Bioassays using woody and herbaceous indicators have been the gold standard method for grape certification programs [6], but these methods have several drawbacks. Asymptomatic responses in indicator plants can occur due to warmer-climate conditions [7] and infection by certain virus variants [8,9]. In addition, bud grafting failure can significantly reduce the number of indicators that are graft-inoculated. Finally, the two to three years required for screening plant materials by the woody index imposes a significant delay in germplasm release to nurseries and growers [8,10,11,12].

Conventional molecular methods like enzyme linked immunosorbent assay (ELISA) and PCR are sensitive but require knowledge of the target pathogen and therefore are not effective at detecting unknown or novel pathogens. In addition, their high specificity can lead to false negative test results for viruses with high genetic diversity [8,10].

High-Throughput Sequencing (HTS), also known as next generation sequencing (NGS), is now broadly used in nearly all areas of plant virus detection. It is demonstrated that HTS targets all nucleic acid types nonspecifically without the need for background knowledge of targeted viruses [13,14,15]. HTS also facilitates detection of known viruses, discovery of novel viruses, and the viruses associated with unknown disease etiology [13,14,16,17,18,19,20,21]. The detection capacity of HTS is independent of environmental conditions, virus genetic variability, and host responses [8]. It is well documented that HTS-based detection is at least equivalent to conventional biological and molecular methods in sensitivity, specificity, repeatability and reproducibility, and surpasses in terms of scalability, data throughput, cost, sensitivity, and specificity for novel and variant isolates [8,10,22]. HTS is the fastest and most cost-effective method for screening plant materials for multiple viruses with a turnaround time of only a few weeks or months [8,15,22]. Therefore, HTS-based diagnostics shortens the time it takes to screen propagation materials from 2–3 years to 9–12 months. Reduced screening time leads to earlier propagation of planting material and a significant reduction in the time required for the final release of virus-tested material [10]. However, HTS is not currently used in certification programs because of the absence of validation criteria required by certifying agencies.

There are unique challenges in developing an HTS-based protocol for plant certification including: (1) the type of plant tissue to sample, (2) viral genomes that can be RNA or DNA, (3) variability of virus titer due to host and environmental factors, and (4) the need to control for possible assay failure.

For this study, we used the molecular assay validation performance criteria of detection sensitivity, specificity, repeatability, and reproducibility [23] to investigate the performance of an HTS-based protocol for the detection of grapevine viruses. Two sampling methods, spring petioles and fall canes, and two extraction methods, TNA and dsRNA, were employed. A protocol for the positive control, *Phaseolus vulgaris* endornavirus 1 (PvEV-1) and 2 (PvEV-2) infecting the common bean cv Black Turtle Soup (BTS), is evaluated. The protocols and performance levels described here may help to standardize HTS for quality assurance and accreditation purposes in plant quarantine or certification programs.

## 2. Materials and Methods

### 2.1. Plants

The 18 infected grapevine selections used in this study (Table 1) are maintained in the Davis Virus Collection [8,24] and are used as virus positive controls by FPS [25]. The 18 vines were selected because they are infected by a broad range of common DNA and RNA viruses and viroids. The vines are maintained in field conditions like many of the samples that are received for diagnostic purposes. One additional vine (cv. ‘Ganzin’) was included in the test panel as the healthy control. This vine was collected from an FPS certified rootstock foundation vineyard. With the exception of a hop stunt viroid (HSVd) infection, the vine has consistently tested negative for more than 40 viral agents. This vine is referred to as a healthy control. All vines were tested by HTS and real-time reverse transcription quantitative PCR (RT-qPCR) or qPCR as described previously [26,27].

### 2.2. Relative Concentration of PvEV-1 and PvEV-2 in Common Bean Cv Black Turtle Soup (BTS) at Different Growth Stages

Six biological replicates of BTS were grown in a greenhouse at 27 °C under 16/8 light/dark regime. From day 10 to day 40, with a 5-day interval, two leaves per plant were collected, combined, and considered as one sample. Each sample was kept at −80 °C until a total of 42 samples were collected. A total of 1 g leaf tissue per sample was homogenized in 10 mL lysis buffer (4 M guanidine isothiocyanate; 0.2 M sodium acetate, pH 5.0; 2 mM EDTA; 2.5% (w/v) PVP-40) using a Homex grinder (Bioreba, Reinach, Switzerland) followed by TNA extraction with the MagMax Plant RNA Isolation kit (ThermoFisher Scientific, Sunnyvale, CA, USA) excluding DNase treatment. TNA was quantified with the Qubit (Invitrogen, Carlsbad, CA, USA). The relative concentration of PvEVs at each five-day interval was measured by RT-qPCR using the TaqMan Fast Virus 1-Step Master Mix kit (ThermoFisher) according to the manufacturer’s protocol. PvEV primers and probes were selected from Kesanakurti et al. [28]. Thermocycler conditions were 5 min at 50 °C, 20 s at 94 °C, and then 40 cycles of 3 s at 94 °C and 30 s at 60 °C.

### 2.3. TNA and dsRNA Extraction

Petioles and dormant canes were collected from the 19 vines in late May and October (spring and fall), respectively. Petioles or cane scrapings (665 mg/sample) were processed and spiked with 35 mg of BTS leaf tissue and then extracted for TNA (Table 2) as described above. The amount of BTS spike-in was determined based on a comparison of 5% or 10% BTS leaf to grape tissue (w/w) (data not shown). TNA quality and quantity were assessed by the Qubit and the Bioanalyzer (Agilent).

Three g of petiole tissue or two g of cane scrapings were spiked with 150 and 100 mg BTS leaf tissue, respectively, and extracted for dsRNA (Table 2) according to the protocol of Kesanakurti et al. [28], excluding DNase and RNase treatments.

### 2.4. Preparation of Dilution Series

Three virus-infected plants (Table 1; Plant ID: 7 [sample set 1], 9 [sample set 2], 15 [sample set 3]) were selected based on the presence of known and novel RNA or DNA viruses from different families and used to create a dilution series. Infected petiole or cane tissue was mixed with tissue from healthy plants in the appropriate percentages to make five dilutions (1%, 2%, 5%, 10%, and 20%) in addition to 100% (Table 3). A total of 665 mg diluted or undiluted tissue was spiked with 35 mg (5% w/w) BTS leaf, ground in buffer, and stored at 4 °C. TNA was extracted twice from the same lysate but on different days. The quality and quantity of extracted TNAs were checked by the Qubit and the Bioanalyzer.

### 2.5. HTS

For individual samples, a total of 700 ng per 10 µL of extracted nucleic acids were subjected to rRNA depletion (only for TNA-based input) and cDNA library construction. Later, cDNA libraries were end-repaired, adapter-ligated by unique dual-indexes, PCR enriched, and used in four separate HTS runs (Table 3). Finally, the amplicons were sequenced in an Illumina NextSeq 500 platform using a single-end 75-bp format. To reduce carryover from the previous run, three washes were performed prior to sample loading. Illumina reads were demultiplexed and adapter trimmed prior to analysis using Illumina bcl2fastq v2.20.0.422.

### 2.6. Determination of an HTS Positive

Trimmed reads were directly mapped to a database using bowtie2 with the—verysensitivelocal option. Reads that could map to multiple locations were placed randomly. The curated database consisted of the GenBank nucleotide sequences for the 29 viruses and viroids tested in this study (Table 1) curated for correct taxonomic attribution plus the reference genome sequences of two positive controls and the *V. vinifera* host (File S1). A sample was considered positive by HTS if more than 10 reads were found mapping to the reference sequences of a viral species using bowtie2. Performance evaluation was restricted to the 29 known grapevine viruses in our curated database.

### 2.7. Characterizing Novel and Non-Grapevine Viruses

Independently, samples were inspected for novel viruses using a SPAdes v3.13 [29] de novo assembly followed by alignment to the National Center for Biotechnology Information (NCBI) GenBank database using BLASTx.

Diagnostic sensitivity, referred to here as just sensitivity, measures the fraction of true virus infections detected by a diagnostic assay. Sensitivity was calculated as the number of true positives detected divided by the total number of PCR-confirmed viruses known to be in the samples [30]. HSVd was not included in the dilution series of performance analyses due to its presence in the healthy vine. Diagnostic specificity, referred to here as just specificity, measures the ability of a diagnostic assay to correctly identify negative results. We characterized the false positive rate in samples from infected and healthy plant tissue (Table 2). For this study, the false discovery rate (FDR), the fraction of positives that are false, was used as the most appropriate measure of specificity. FDR was estimated using the formula: false positives detected/total positives detected [30].

A diagnostic assay’s precision is the extent to which repeated testing on identical plants renders consistent results, both in a short time-span and within the same run (repeatability) and over a longer time span between independent runs (reproducibility) [23,31]. In this study, repeatability was assessed by comparing the results from replicate extractions of sample sets 1 and 3 (Table 3). The reproducibility of the assay was assessed from independent extractions of two tissue types, spring petioles and fall canes, from all three sample sets (Table 3). Following a standard procedure for qualitative assays, the results from these comparisons are summarized in contingency tables (Table S3) from which agreement measures and kappa statistics were calculated [23].

## 3. Results

### 3.1. Concentration of PvEV-1 and PvEV-2 in BTS at Different Growth Stages

**Figure 1:**
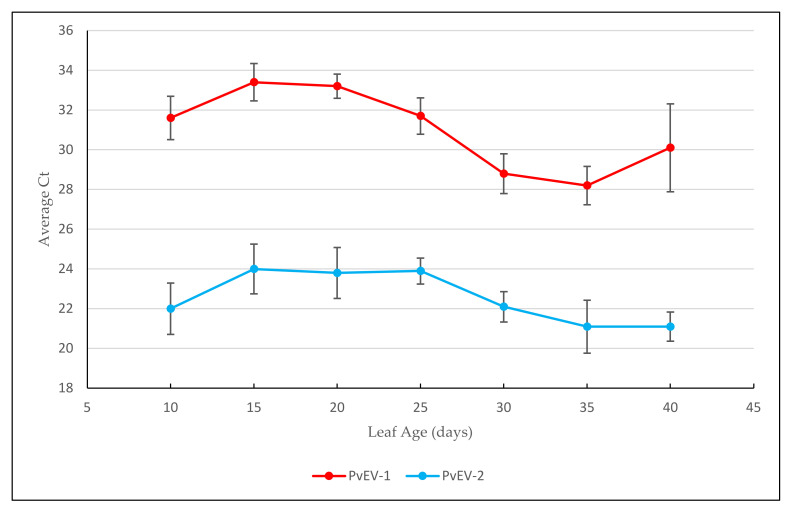
Average Ct values of endornaviruses PvEV-1, PvEV-2 from extracted TNA of common bean leaves over a 40-day developmental period. The bars indicate standard error.

A 40-day trial was conducted on six BTS biological replicates to assess the effect of leaf developmental stage on the average cycle threshold (Ct) values of PvEV-1 and PvEV-2. The Ct value averages ranged from 28.2–33.4 for PvEV-1 and 21.1–24.0 for PvEV-2 over the trial period (Figure 1). Within each virus, there was no significant difference (*p* < 0.05) in average Ct values over a 40-day growth period.

### 3.2. Evaluating BTS as a Positive Control

Raw data from all HTS runs showed an average of 25.7 million sequences with an average coefficient of variation of 12.4% (Table S1). To determine the efficiency of BTS spike-in to samples of HTS run 2 (Table 2) and to track PvEV as the internal positive control, correlation analysis between either PvEV-1 or PvEV-2 and total read number of individual samples was conducted. The analysis revealed a significant (*p* < 3.2 × 10^−5^) positive correlation between total read number of individual samples and read number of PvEV-1 (R^2^ = 0.66) or PvEV-2 (R^2^ = 0.62). Comparing BTS free fall canes to BTS spiked spring petioles showed a slight decrease in sensitivity from 98% to 97% and a slight increase in FDR from 17% to 18%. Both PvEV-1 and PvEV-2 were detected in all 129 samples analyzed in seven independent HTS runs (Table 1 and Table 2, Table S2). The proportion of PvEV-1 reads in TNA samples ranged between 0.01–0.05% of the total reads with a genome coverage of 97.1–99.9%. The proportion of PvEV-2 reads ranged between 0.01–0.05% of the total reads with a genome coverage of 96.7–99.9%. In the dsRNA samples, we discovered 13 low coverage outliers described in detail below. Excluding these outliers, read counts were higher for dsRNA. The proportion of PvEV-1 reads ranged between 0.5–5.5% of the total reads with a genome coverage of 99.9–100%, and the PvEV-2 reads ranged between 0.5–8.8% of the total reads with a genome coverage of 99.7–100%.

### 3.3. Evaluation of Our TNA HTS Protocol

Initially, we evaluated our TNA HTS protocol on both spring petioles and fall canes from the complete set of 19 vines (Table 2). From that, we estimated the sensitivity and specificity of the assay on the broadest set of viruses in this study. Out of a combined total of 264 PCR-verified viruses and viroids, 258 were detected as true positives, resulting in an overall sensitivity of 98%. All 51 viroid infections were detected in both spring petiole and fall cane for a sensitivity of 100%. Sensitivity for detecting viruses in fall canes (98%) was slightly higher than for spring petioles (95%). The six false negatives were from four virus families that included both RNA and DNA genomes. ArMV was not detected in fall canes twice, and GRGV, GBV-1, GAMaV, and GLRaV7 were not detected in spring petioles. In all of these cases, if the virus was not detected in the spring petiole sample, it was detected in the fall cane sample from the same grapevine, and vice versa.

Specificity was measured using FDR. There was a total of 132 false positives, resulting in an average FDR of 21%. In other words, approximately 1 in 5 positives was false.

Finally, we observed a range of mycoviruses for all grapevines in the BLAST results from the de novo assemblies (data not shown), underscoring the broader ability of HTS to detect viruses [8,32].

### 3.4. Comparison to dsRNA

Parallel dsRNA extractions, library preparation, and sequencing were performed for all 38 spring petiole and fall cane samples from the 19 grapevines (Table 2). Examination of the PvEV-1 and PvEV-2 positive control viruses revealed 13 samples with fewer than 1000 reads for both viruses (Table S2). These read counts were outliers and below the threshold established in Kesanakurti et al. [28] and below the lowest read counts obtained from the TNA samples. Out of caution, all 13 samples along with their corresponding TNA samples were removed from the side-by-side performance comparison. This removed grapevines 2, 9, and 18 completely. The remaining 25 samples from 16 grapevines were compared.

Average read count for the 99 PCR-verified distinct virus infections was 3.2 times higher for dsRNA than TNA, 98,000 reads vs. 30,000. However, the diagnostic sensitivity was close between the two extraction methods when only viruses were considered. The dsRNA extraction method had one false negative for an estimated sensitivity of 99%. The TNA extraction method had two false negatives for an estimated sensitivity of 98%. Both missed an ArMV positive in cane 17. A GRGV positive in petiole 12 was missed by TNA.

The dsRNA extraction method was much less sensitive for viroids in fall canes. In fall canes, 15 out of 38 viroid infections were missed in dsRNA samples while none were missed in TNA samples. In spring petioles, none of the 29 viroid infections were missed by dsRNA or TNA. This changed the overall sensitivity to 91% for dsRNA and 98% for TNA. The FDR for all viruses/viroids was 25% for dsRNA and 17% for TNA (Table S4).

### 3.5. Dilution Series

**Figure 2:**
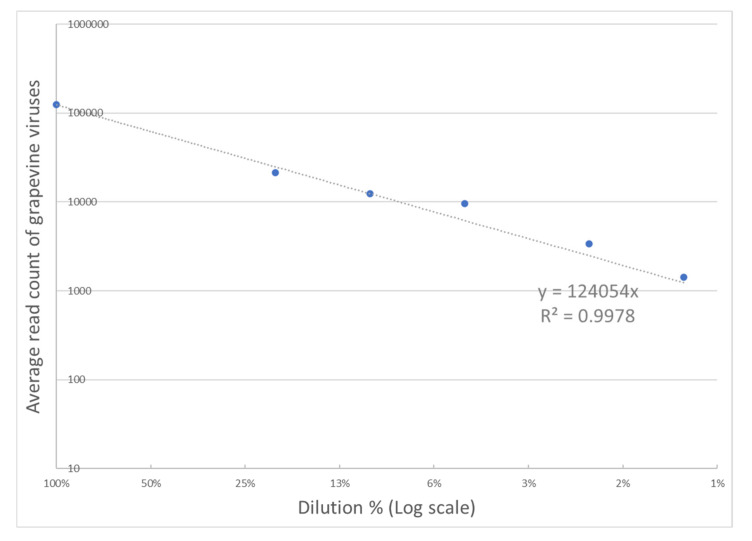
Impact of dilutions on grapevine virus read counts. The average count of reads mapped to grapevine virus sequences is averaged for and plotted against dilution. Regression through the origin demonstrates a high degree of linearity (R^2^ > 0.99).

For a more detailed performance analysis of the TNA-based HTS assay over a broad range of virus concentrations in three infected vines, replicate dilutions of infected tissue into healthy tissue down to 1% were prepared and sequenced. The number of mapped reads for all grapevine viruses/viroids varied linearly with dilution over the full range (Figure 2; R^2^ > 0.99). Only one virus, GLRaV-7, showed read counts that were inconsistent with dilution (Figure S1).

Average sensitivity across all dilutions, tissue types, and sample sets was 95% (Table 4 and Table S3). Breaking out sensitivity by dilution, average sensitivity ranged from a high of 100% for the undiluted samples down to 98% for the 5% dilution. The two lowest dilutions registered a more substantial drop in sensitivity with 93% for the 2% dilution and 85% for the 1% dilution. Looking at spring canes and fall petioles separately, overall sensitivity was slightly higher for petioles (97%) compared to canes (94%).

We investigated further the effect of virus concentration on the detection of specific viruses in each of the three sample sets (Table S3). In sample set 1, there were ten viruses/viroids including GLRaV-2, GLRaV-4, GRSPaV, GRVFV, GVA, GVB, GVD, GVF, GYSVd-1, and GYSVd-2. In the cane tissue, eight viruses were detected in all dilutions, but the limit of detection for two viroids, GYSVd-1 and GYSVd-2, was the 20% and 5% dilutions, respectively. In the petiole tissue, seven pathogens were detected down to the 1% dilution, but the limit of detection for GRVFV and GVD was the 5% dilution, and, for GYSVd-1, the 2% dilution. In this sample set, the average sensitivity of detected pathogens over infection dilutions in the petiole tissue was higher than the cane tissue. In sample set 2, there were ten viruses/viroids including AGVd, GLRaV-1, GLRaV-7, GRSPaV, GRVFV, GVA, GYSVd-1, GYSVd-2, and two recently detected novel viruses, GKSV and GPoV-1 [33,34]. In the cane tissue, seven viruses were detected down to the 1% dilution; however, the three viroids were only detected down to the 2% dilution. In the petiole tissue, nine pathogens were detected down to the 1% dilution, with the exception of GLRV-7. For GLRaV-7, an average of 158,000 read counts were obtained from the extracted undiluted cane tissues; however, there was only an average of 16 read counts from the extracted undiluted petiole tissues. As a result, the average sensitivity over all dilutions for sample set 2 was higher in cane than petiole tissue. In sample set 3, there were seven viruses/viroids including GFkV, GLRaV-2, GRBV, GRSPaV, GRVFV, GVB, and GYSVd-1. All seven pathogens in both cane and petiole tissues were detected down to the 1% dilution.

The overall FDR was 21% (Table S3). FDR was negatively correlated with dilution, ranging from 32% for the undiluted samples to 13% for the 1% dilution. Overall, FDR was slightly lower for petioles (16%) compared to canes (22%).

Four false positives were selected to investigate if the source was in the extracted TNAs or the subsequently constructed libraries. These samples were (i) 20% diluted petioles of sample set 2 with a false positive for GLRaV-2 (read count [RC]: 167), (ii) 1% diluted dormant canes of sample set 1 with a false positive for GLRaV-7 (RC: 70) in, (iii) undiluted dormant canes of sample set 2 with a false positive for GLRaV-2 (RC: 315), and (iv) 10% diluted dormant canes of sample set 2 with a false positive for GLRaV-2 (RC: 205) (Table S3). (RT)-qPCR results showed that the extracted TNA was negative for the corresponding viruses in all four cases. However, in the cDNA libraries, samples “ii”, “iii”, and “iv” tested positive and sample “i” tested negative for the respective viruses.

### 3.6. Repeatability

Repeatability was assessed by intra-run comparison of replicate extractions from the same lysate in sample sets 1 and 3 (Table 4). Repeatability over all data was 98% (Table S3) with a Cohen’s kappa of 0.95 corresponding to almost perfect agreement [35]. Petioles had slightly higher repeatability (99%) compared to canes (97%). Repeatability was also measured by dilution. For dilutions from 100% down to 5%, repeatability ranged from 98% to 100% with no apparent correlation with dilution. For the two lowest dilutions of 2% and 1%, repeatability was measured at 96%.

### 3.7. Reproducibility

Two independent TNA extractions of spring petiole and fall cane tissue in all three sample sets were used for estimating reproducibility. As expected, reproducibility (comparing spring canes to fall petioles) was lower than repeatability. Reproducibility over all data was 91% with a Cohen’s kappa value of 0.81, at the lower end of “almost perfect agreement” [35]. Examining the effect of dilution, reproducibility ranged from 92% to 94% in dilutions down to 5% with no apparent correlation with the dilution level and all kappa values falling in the range of almost perfect agreement (Table S3). For the two lowest dilutions of 2% and 1%, reproducibility was measured at 90% and 86%, respectively, with kappa values falling in the range of substantial agreement.

## 4. Discussion

Limiting the spread of grapevine viruses requires screening planting material to ensure it is free of harmful viruses. It is documented that HTS-based detection can perform at least as well or better than conventional biological and molecular methods in sensitivity, specificity, repeatability, and reproducibility, and surpasses them in terms of scalability, data throughput, cost, sensitivity, and specificity for novel and variant isolates [8,10,22]. Several studies have investigated different aspects of HTS performance criteria, including different extraction protocols and sequencing platforms [36,37], independent bioinformatic pipelines [30,37,38], and different nucleic acid templates [19,37,39]. In this study, we evaluate a TNA HTS-based detection assay for screening purposes. We identified the viruses present in a large pool of 19 grapevines that were PCR-positive for a wide range of viruses. We evaluate the performance of our diagnostic assay by measuring sensitivity and specificity. We evaluated two tissues: spring petioles and fall canes. We evaluate the contribution of the extraction method to detection using a side-by-side comparison to dsRNA. Additionally, we estimated repeatability, reproducibility, and evaluated the limits of detection for a subset of viruses using a dilution series. We also describe and evaluate a protocol for a positive control for virus identification using a TNA HTS protocol.

Inclusion of a positive control at the start of sample processing can help detect false negatives due to assay failure. It may also serve as a quantitative threshold for detection performance [28]. In the Kesanakurti et al. study, several fruit tree and grapevine samples were spiked with BTS 50 mm^2^ leaf discs and extracted for dsRNA. In that study, the proportion of PvEV-1 reads to total read counts of a sample ranged between 0.1% to 1% for dsRNA. In this study, the proportion of PvEV-1 and PvEV-2 reads from extracted TNA samples ranged between 0.01–0.05%. As expected, both studies observed a significant positive correlation of the read count to the total read number of each sample. Despite the lower number of reads, near-complete PvEV genome coverage (>96.7%) was observed in our TNA extractions. Side by side comparison of BTS-free fall canes to BTS-spiked spring petioles showed a slight decrease in sensitivity (1%) and a slight increase in FDR (1%). While we cannot directly attribute this to BTS, it was in contrast to the general trend observed for canes and petioles in the dilution series. Both PvEV-1 and PvEV-2 were detected in all 129 samples analyzed in seven independent HTS runs. The lower proportion of PvEV reads retrieved from TNA in the current study (0.01–0.05%) versus dsRNA from the Kesanakurti et al. study is most likely due to the difference in extraction efficiency that resulted in higher PvEV concentrations. Our results suggest a BTS spike-in for TNA template may be employed as an effective internal positive control to ensure the reliability of multiple stages of an HTS protocol. Additional work should be done to further quantify and optimize the effects of the positive control on sensitivity and specificity. In addition, for ensuring a specific detection threshold, further work can be done to quantify the appropriate amount to introduce into the sample.

For HTS-based detection, several nucleic acid templates have been used, including small RNA (sRNA), total RNA, TNA, and dsRNA [13]. Since viruses infecting grapevine have either RNA or DNA genomes and viroids have RNA genomes, the use of TNA allows the detection of both nucleic acid types [14,40,41]. Since plants do not contain high molecular weight dsRNA, and dsRNA is formed during replication of RNA and DNA viruses [42,43], it can be utilized as an enriched source for virus detection [8,28,44]. Our comparison of dsRNA and TNA indicates that sensitivity is lower for dsRNA due to the high rate of false negatives for viroids in fall canes, which lowered its overall sensitivity to 91% compared to 98% for TNA. On viruses, sensitivity of the dsRNA extraction method was slightly higher than TNA. Specificity was also lower for TNA compared to dsRNA, which may be the result of the virus sequence enrichment and greater stability of the dsRNA molecules. Practically speaking, dsRNA preparation is more time-consuming than TNA, three days compared to several hours; has lower testing scalability; and increases cross-contamination risk given its higher stability in the environment [45]. In addition, we observed 13 unexplained negative outliers for the positive control when using dsRNA compared to TNA. These results suggest that TNA should be considered as a viable and attractive alternative to dsRNA in consideration of the higher sensitivity and specificity and some of the practical disadvantages associated with dsRNA templates.

We included both spring petioles and fall canes in all of our evaluations to account for possible false negative test results due to variable virus titer and uneven within-vine distribution. These issues have been documented with GLRaV-1, GLRaV-2, GLRaV-3 [46,47,48,49], GVA, GVB, GRSPaV [50], GFLV [51], and GRBV [52]. In the initial broad evaluation of our HTS-based assay, we observed six false negatives when using TNA extracted from spring petioles and fall canes (Table S4). These false negatives included RNA and DNA viruses from four families: two each from *Secoviridae*, and *Tymoviridae*, and one each from *Caulimoviridae* and *Closteroviridae*. In all cases, the spring petiole false negatives were detected in the fall cane samples and vice versa. It seems likely that these false negative results were the result of lower virus titer that may have been exacerbated by seasonal and tissue effects. The apparent lack of correlation in the false negatives for spring petioles and fall canes in our data suggests that the two test results can be combined for greater sensitivity by counting a positive from either test as a positive for the plant. Under this scenario, sensitivity increases to 100%, even in our dilution series down to 1% for viruses and down to 2% for viroids.

Because virus titer can be highly variable, we further evaluated HTS detection sensitivity and determined the limit of detection by analyzing a dilution series of three infected grapevine samples (Table 3) diluted with a healthy control down to 1%. The limit of detection for the viruses was lower (better) for fall cane tissues, down to 1% dilution. In the case of GLRaV-7, it was detected consistently in fall cane samples, but only in the undiluted spring petiole samples. Most likely because the petioles collected in spring had a low titer of the virus, as the Ct showed a high value of 29.6 (data not shown). This situation is similar to other leafroll viruses that have higher titer in the late growing season [53,54]. Likewise, virus distribution within the plant should also be considered for the inconsistency of GLRaV-7 detection.

In the dilution series, average read count for the majority of viruses/viroids monotonically decreased with dilution (Figure 2 and Figure S1), which was consistent with studies utilizing sRNA, dsRNA, and ribo-depleted total RNA templates that observed depth reduction resulted in decreased sensitivity [30,36,39]. Averaging over dilutions from 100% through 5%, sensitivity for virus/viroids was measured at 99% with no individual dilution dropping below 98%. However, there was a notable decrease in sensitivity at the two lowest dilutions. Averaging over the entire dilution series, sensitivity drops to 95%.

### 4.1. Sensitivity

For plant certification, sensitivity is the most important performance criteria for a diagnostic assay. The current study demonstrates the comprehensiveness of HTS for the detection of numerous viruses/viroids reflecting the results of several other studies that have used different input templates including total RNA and sRNA [19], dsRNA [8,22], sRNA [17,30], dsRNA and total RNA [44], dsRNA, total RNA, and sRNA [39], and TNA [14,40]. Overall sensitivity for our TNA HTS assay in the broad evaluation of 19 vines was 98%. In the dilution series, average sensitivity was 99% averaging over the dilutions down to 5%. Sensitivity was increased to 100% in both experiments by combining spring petiole results with fall cane results. The high degree of linearity to the rate at which viral reads are recovered suggests that additional sequencing is yet another approach to increased sensitivity. In Gaafar and Ziebel study [39], higher sequencing depth was needed to detect pea necrotic yellow dwarf virus, a DNA virus, and a potato spindle tuber viroid from extracted dsRNA. Sequencing depth can be increased, either by reducing the number of samples per HTS run or by using one of Illumina’s higher throughput instruments. It was notable that sensitivity could be increased to 100% by relaxing the threshold to a single read.

### 4.2. Specificity

This measures the ability of a diagnostic assay to correctly identify negative results. Using an HTS-positive threshold of >10 read counts, the FDR was measured at 21% in the broad survey of 19 grapevines and 19% for the dilution series. Practically speaking, this means that about one in five positive test results will be false. This high FDR was the expected tradeoff for increasing sensitivity by employing local sequence alignment and reducing the minimum read count for a positive. It should be noted that changing the read count threshold to detect more or fewer viruses will also change the FDR. A higher FDR rate (lower specificity) is a recognized tradeoff when higher sensitivity is desired [55]. Specifically, our choice of threshold enabled us to avoid false-negative detections such as GLRaV-7. Filtering taxonomically misannotated sequences deposited in GenBank was also useful in reducing the number of false positives. Ultimately, a secondary confirmation method such as PCR could be used to verify the actual presence or absence of an HTS positive in tested samples.

Introduction of erroneous viral agents into a sample arises bioinformatically [30], from cross-contamination during sample or library preparation, as well as index-hopping or sequence carryover during the HTS sequencing process [56]. For the four cases investigated, the initial TNAs tested negative by PCR for the respective viruses but were positive in DNA libraries for three out of four cases. Thus, library preparation can be determined as the source of false positive viruses for those samples. The only case that was negative for both TNA and DNA templates was the sample with false positive results for GLRaV-2, which likely arose by another mechanism.

### 4.3. Repeatability and Reproducibility

These measure the extent to which repeated testing on identical plants renders consistent results, both in a short time-span and within the same run (repeatability) and over a longer time span between independent runs (reproducibility). Virus infections in fall canes, in contrast to spring petioles, were repeatedly detected down to a 1% dilution. Viroid infections in spring petioles, in contrast to fall canes, were repeatedly detected down to dilutions of 2% and 1%. Repeatability over all data in the dilution series was 98% (Table S3) with a Cohen’s kappa of 0.95 corresponding to almost perfect agreement [35].

Detection of viral agents from separate intra-lab extractions of spring petioles paired with fall canes from the same grapevine was used as a measure of reproducibility. Our reproducibility measure captures more variation (season, tissue, extraction, and HTS run) than repeatability, and was therefore expected to be lower. Reproducibility over all data was 91% with a Cohen’s kappa value of 0.81, at the lower end of “almost perfect agreement” [35]. As with repeatability, there was a marked drop off below the 5% dilution. Reproducibility for dilutions from 100% to 5% was 93% (Table S3). This rate is on par with the inter-lab reproducibility rate of 91.6% for an sRNA-based HTS study [30].

## 5. Conclusions

HTS is becoming a routine diagnostic platform for the detection of grapevine viruses/viroids, but evaluating the performance of HTS-based protocols is necessary prior to acceptance and implementation by plant quarantine and certification programs. In this study, we determined the performance characteristics of our TNA HTS assay for the detection of grapevine viruses/viroids. For our TNA HTS protocol, average sensitivity was 98% measured in a broad survey and in a dilution series down to 5%. Average specificity measured by FDR was approximately one in five positives. Two measures of precision, repeatability and reproducibility, were 99% and 93%, respectively. These are both considered in the range of almost perfect agreement using Cohen’s kappa statistic.

Our results suggest that sensitivity can be increased by combining the results from two independent tests of the same plant. Specifically, results from spring petioles were compared to fall canes. While alone spring petioles had slightly better performance than fall canes, combining the two tests resulted in 100% sensitivity. Multiple sampling may be an effective way to overcome documented seasonal and tissue effects in grapevine virus titer.

We believe that these types of performance evaluations will be important in furthering the acceptance of HTS-based diagnostic assays in plant quarantine and certification programs.

## Figures and Tables

**Table 1:** Viruses or viroids detected in selected vines collected from Davis Virus Collection and FPS foundation vineyard collections.

Plant ID	AGVd	ArMV	GAMaV	GBV-1	GEV-1	GFkV	GFLV	GKSV	GLRaV-1	GLRaV-2	GLRaV-3	GLRaV-4	GLRaV-7	GPoV-1	GRBV	GRGV	GRLDaV	GRSPaV	GRVFV	GVA	GVB	GVD	GVE	GVF	GVL	GYSVd-1	GYSVd-2	HSVd	satGVV
**1**					x					x	x	x						x								x		x	
**2**									x		x							x		x	x				x	x	x	x	x
**3**											x	x						x	x							x		x	
**4**	x											x						x	x							x	x	x	
**5**	x										x	x						x	x					x		x	x	x	
**6**	x			x							x	x					x	x		x	x			x		x	x	x	
**7**										x		x						x	x	x	x	x		x		x	x	x	
**8**			x				x				x								x									x	
**9**	x							x	x				x	x				x	x	x						x	x	x	
**10**	x										x							x	x	x						x	x	x	
**11**											x							x					x			x	x	x	
**12**						x				x						x		x			x					x		x	
**13**																		x								x		x	
**14**									x		x	x						x		x				x		x		x	
**15**						x				x					x			x	x		x					x		x	
**16**												x						x								x	x	x	
**17**		x	x																							x		x	
**18**		x	x																							x		x	
**HC**																												x	

AGVd: australian grapevine viroid; ArMV: arabis mosaic virus; GAMaV: grapevine asteroid mosaic-associated virus; GBV-1: grapevine badnavirus-1; GEV-1: grapevine enamovirus-1; GRLDaV: grapevine roditis leaf discoloration-associated virus; GFkV: grapevine fleck virus; GFLV: grapevine fanleaf virus; GKSV: grapevine kizil sapak virus; GLRaV-1: grapevine leafroll-associated virus-1, GLRaV-2, GLRaV-3, GLRaV-4, and GLRaV-7; GVA: grapevine virus A, GVB, GVD, GVE, GVF, GVL; GPoV-1: grapevine poleovirus-1; GRBV: grapevine red blotch virus; GRGV: grapevine red globe virus; GRSPaV: grapevine rupestris stem pitting-associated virus; GRVFV: grapevine rupestris vein-feathering virus; GYSVd-1: grapevine yellow speckle viroid-1, and GYSVd-2; HSVd: hop stunt viroid; satGVV: grapevine satellite virus; HC: healthy control.

**Table 2:** Description of the HTS runs used in a broad evaluation of both TNA and dsRNA extraction methods on both spring canes and fall petioles. Where indicated, common bean cv Black Turtle Soup (BTS) was added as a positive control.

HTS Run	Tissue	No. VP ^a^	No. VN ^b^	Template	Spike-In
1	Cane	18	1	TNA	-
2	Petiole	18	1	TNA	BTS
3	Cane/Petiole	9/9	0/1	dsRNA	BTS
4	Cane/Petiole	9/9	1/0	dsRNA	BTS

^a^: virus positive samples; ^b^: virus negative samples.

**Table 3:** Description of the dilution series HTS runs. Three sample sets were divided into four runs. Each sample set consists of extracts from undiluted virus-infected spring petioles and fall canes and their corresponding dilutions of 20%, 10%, 5%, 2%, and 1%. All extractions were prepared in replicate for a total of 24 samples within each sample set. Sample set 1 = vine 7; sample set 2 = vine 9; sample set 3 = vine 15.

HTS Run #	Tissue	Template	Spike-In	No. Samples per Run	Sample Description
1	Petiole	TNA	BTS	18	Sample set 1, Extraction 1 Sample set 1, Extraction 2 Sample set 2, Extraction 1
2	Cane	TNA	BTS	18	Sample set 2,Extraction 2 Sample set 3, Extraction 1 Sample set 3, Extraction 2
3	Petiole	TNA	BTS	18	Sample set 2, Extraction 2 Sample set 3, Extraction 1 Sample set 3, Extraction 2
4	Cane	TNA	BTS	18	Sample set 1, Extraction 1 Sample set 1, Extraction 2 Sample set 2, Extraction 1

Four performance characteristics of an HTS-based diagnostic assay.

**Table 4:** Sensitivity for dilutions of three virus-infected grapevine sample sets comprised of two tissue types extracted in replicate.

Infection Dilution (%)	Detection Sensitivity (%)						Average
	Sample Set 1		Sample Set 2		Sample Set 3		
	Cane	Petiole	Cane	Petiole	Cane	Petiole	
100	100	100	100	100	100	100	100
20	95	100	100	90	100	100	98
10	90	100	100	90	100	100	98
5	90	100	100	90	100	100	98
2	85	80	100	90	100	100	93
1	80	70	70	90	100	100	85
Average	90	92	95	92	100	100	95

## Data Availability

The data presented in this study are available in the current article or Supplementary Materials.

## Supplementary Materials

The following are available online at https://www.mdpi.com/article/10.3390/v13061130/s1, Figure S1: Average read counts of respective viruses over infection dilutions of grapevine samples, File S1: Curated database of 29 viruses used in this study for mapping stage of bioinformatic analysis, Table S1: Total read counts of the entire samples from HTS runs, Table S2: Evaluation of BTS endornaviruses (PvEV-1, PvEV-2) read counts and genome coverage from extracted TNA and dsRNA across the entire HTS runs, Table S3: Viruses and viroids detected in infection dilutions of extracted TNAs from three sample sets, Table S4: Detected viruses and viroids from dsRNA and TNA extraction of both cane and petiole of nineteen vines.
