# Supplementary material for: Quality Assessment and Validation of High-Throughput Sequencing for Grapevine Virus Diagnostics
Source: Viruses. 2021 Jun 11;13(6):1130. doi: 10.3390/v13061130 (PMC8231206; doi:10.3390/v13061130)
Supplement: Supplementary file 1 [file viruses-13-01130-s001.zip › Fig. S1.pdf]

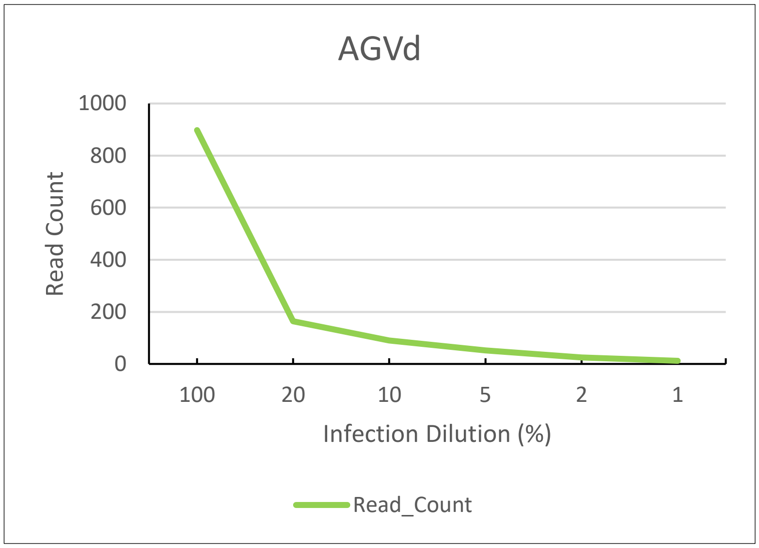
AGVd
Read Count
1000
800
600
400
200
0
100
20
10
5
2
1
Infection Dilution (%)
Read_Count

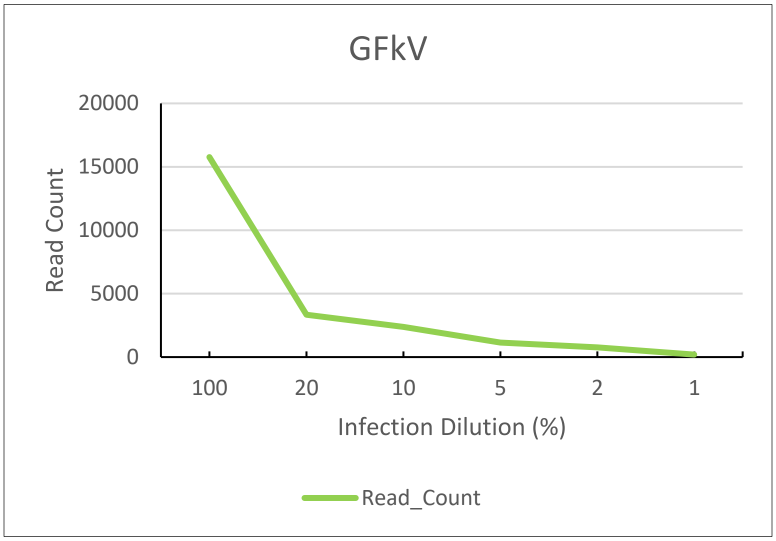
GFkV
Read Count
20000
15000
10000
5000
0
100
20
10
5
2
1
Infection Dilution (%)
Read_Count

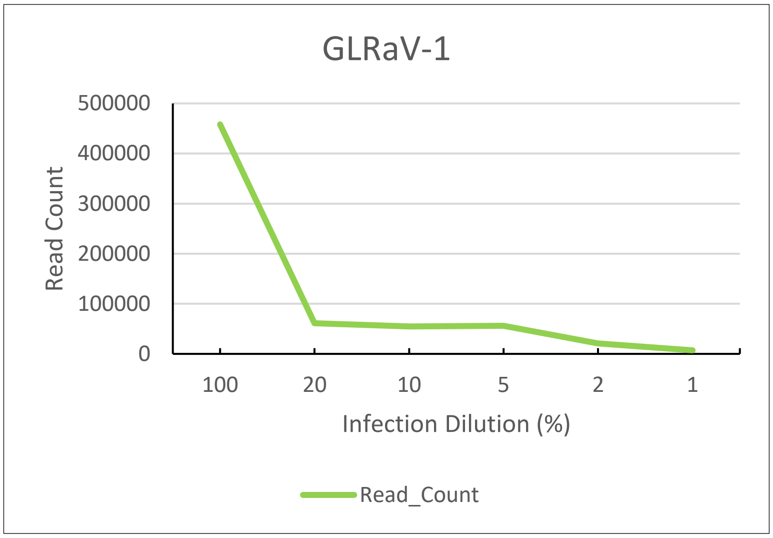
GLRaV-1
Read Count
500000
400000
300000
200000
100000
0
100
20
10
5
2
1
Infection Dilution (%)
Read_Count

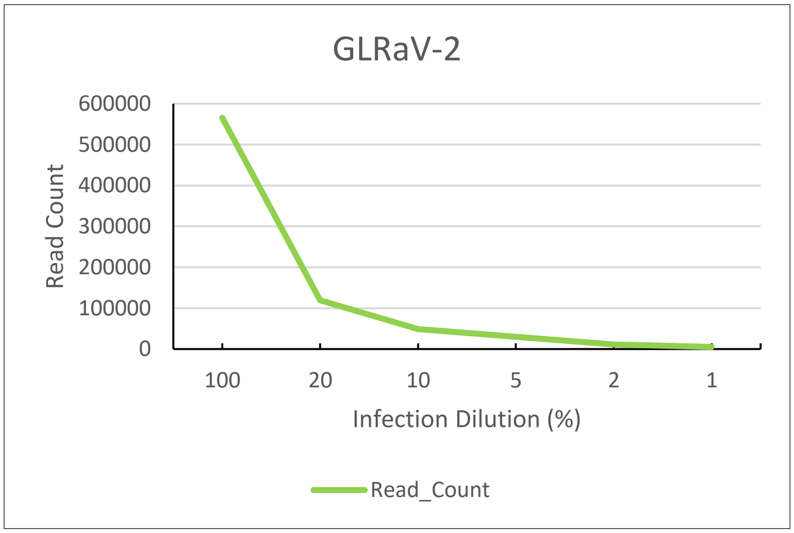
GLRaV-2
Read Count
600000
500000
400000
300000
200000
100000
0
100
20
10
5
2
1
Infection Dilution (%)
Read_Count

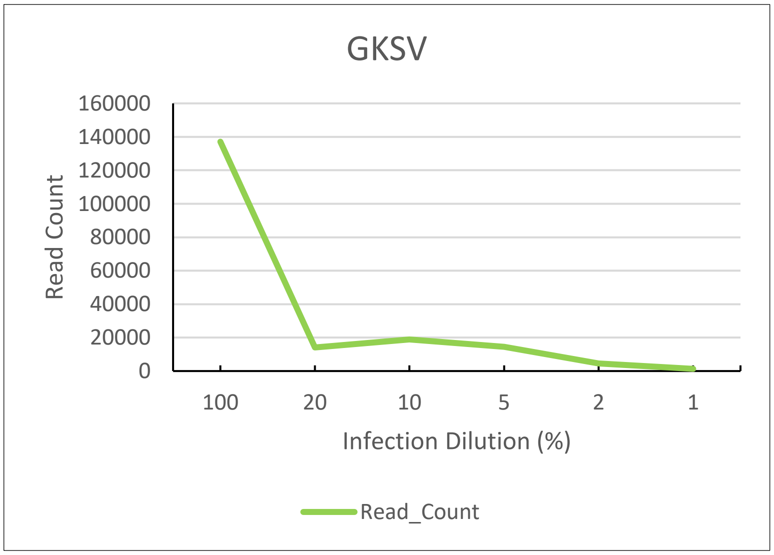
GKSV
Read Count
160000
140000
120000
100000
80000
60000
40000
20000
0
100
20
10
5
2
1
Infection Dilution (%)
Read_Count

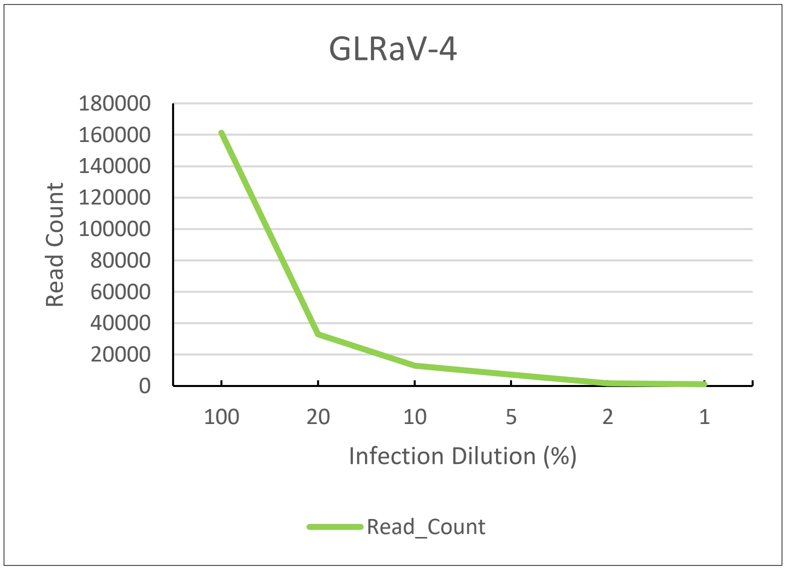
GLRaV-4
Read Count
180000
160000
140000
120000
100000
80000
60000
40000
20000
0
100
20
10
5
2
1
Infection Dilution (%)
Read_Count

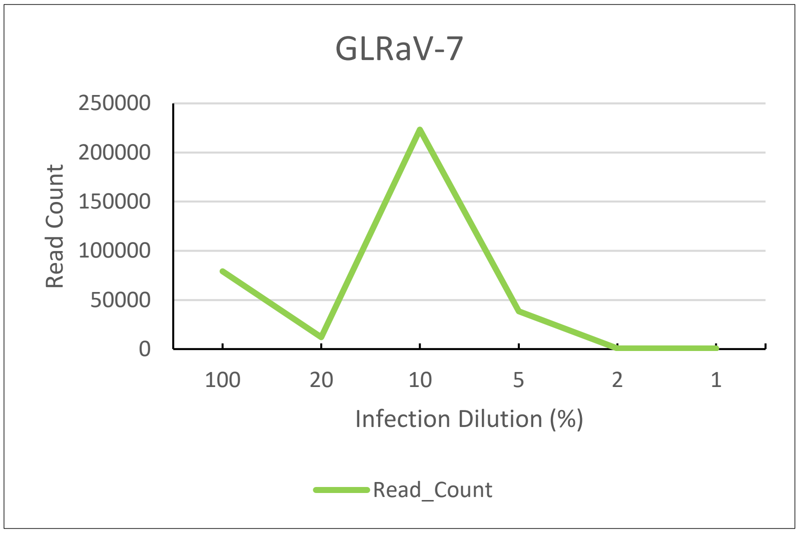
GLRaV-7
Read Count
250000
200000
150000
100000
50000
0
100
20
10
5
2
1
Infection Dilution (%)
Read_Count

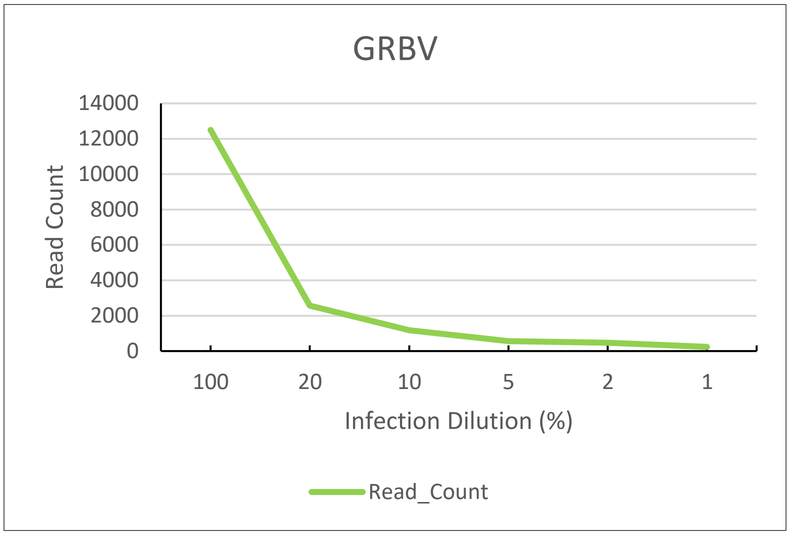
GRBV
Read Count
14000
12000
10000
8000
6000
4000
2000
0
100
20
10
5
2
1
Infection Dilution (%)
Read_Count

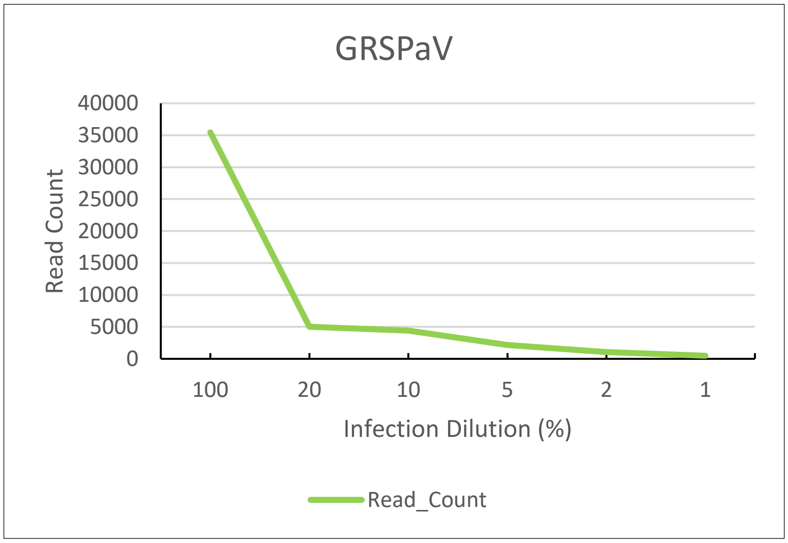
GRSPaV
Read Count
40000
35000
30000
25000
20000
15000
10000
5000
0
100
20
10
5
2
1
Infection Dilution (%)
Read_Count

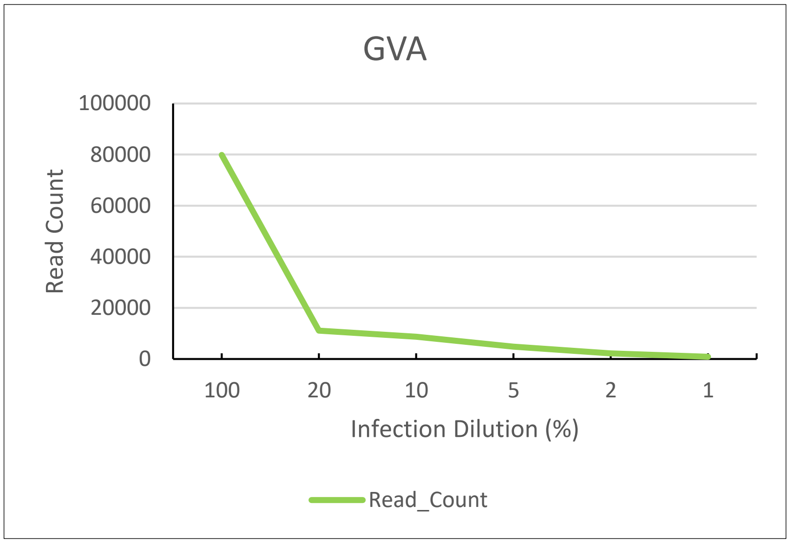
GVA
Read Count
100000
80000
60000
40000
20000
0
100
20
10
5
2
1
Infection Dilution (%)
Read_Count

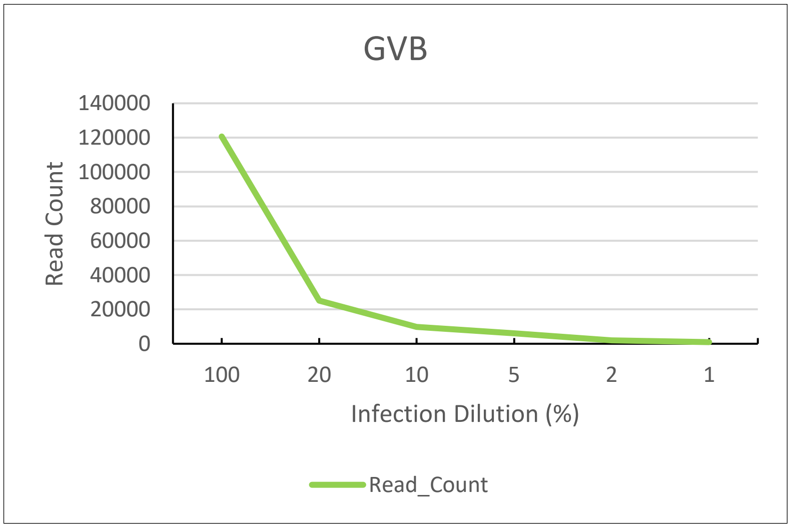
GVB
Read Count
140000
120000
100000
80000
60000
40000
20000
0
100
20
10
5
2
1
Infection Dilution (%)
Read_Count

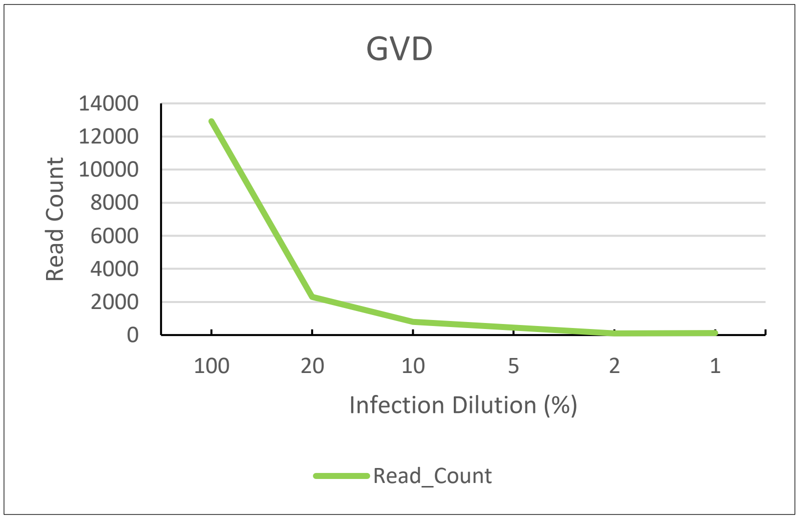
GVD
Read Count
14000
12000
10000
8000
6000
4000
2000
0
100
20
10
5
2
1
Infection Dilution (%)
Read_Count

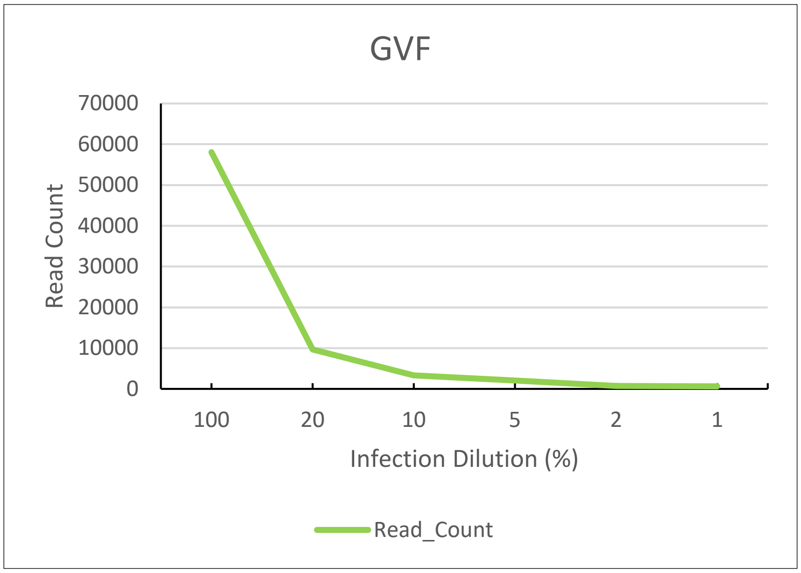

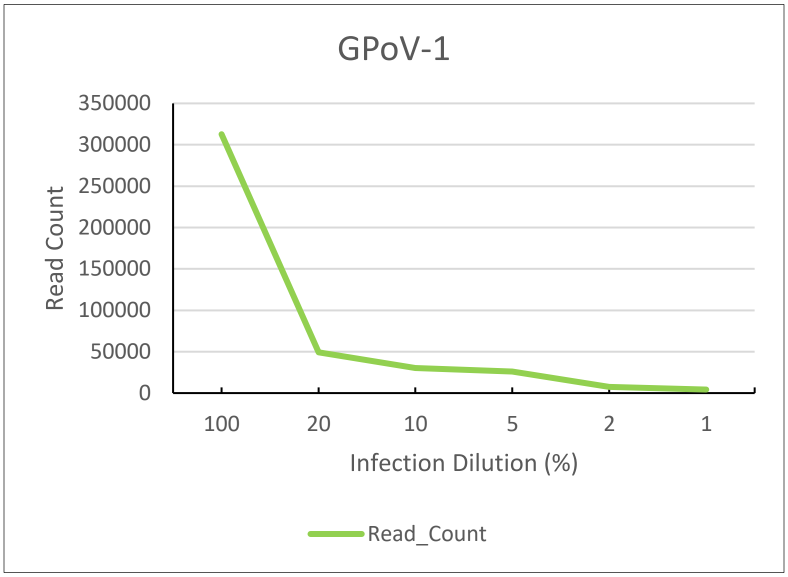

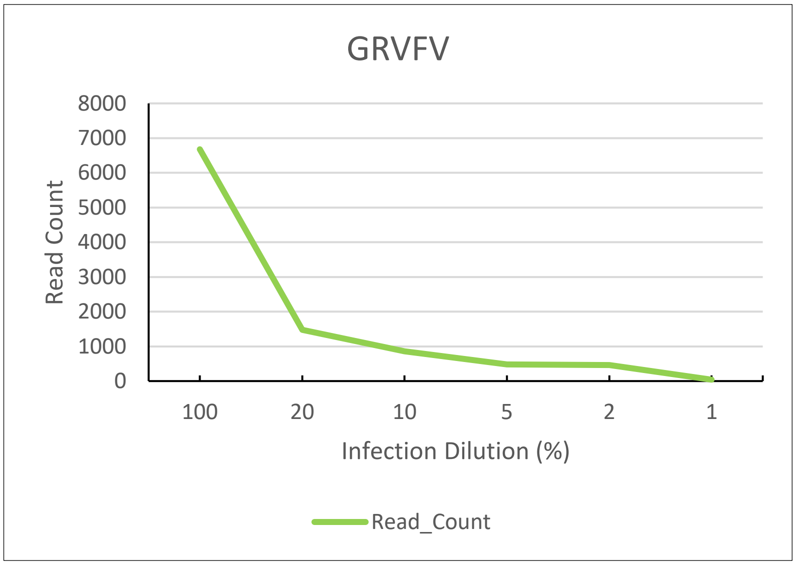

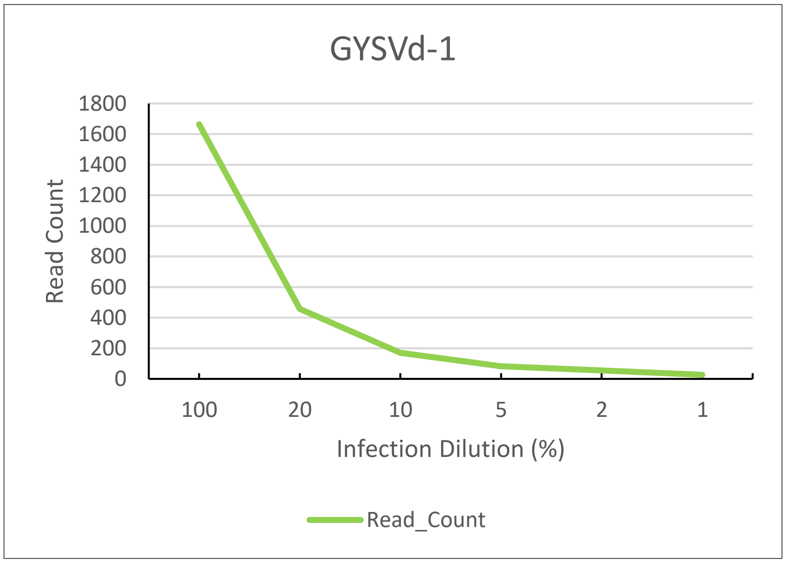

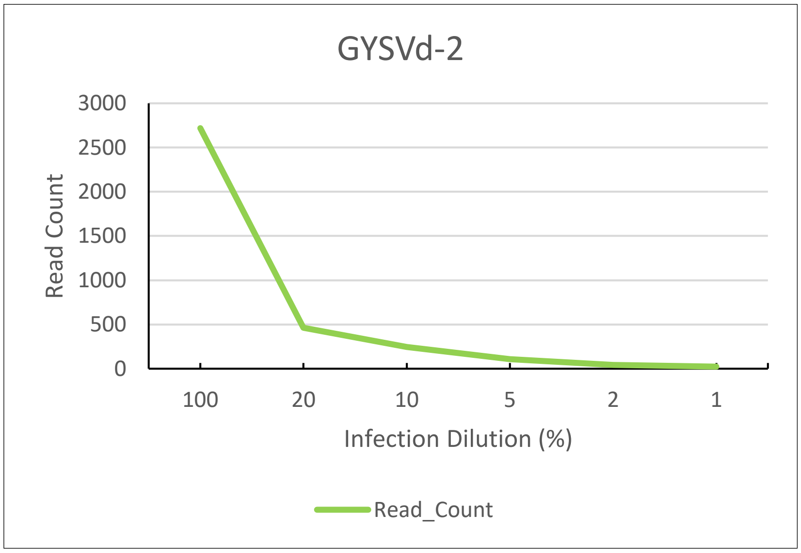

Fig. S1. Average read counts for respective viruses and viroids over infection dilutions of grapevine samples sets.
